# DNA Damage Responses during the Cell Cycle: Insights from Model Organisms and Beyond

**DOI:** 10.3390/genes12121882

**Published:** 2021-11-25

**Authors:** Delisa E. Clay, Donald T. Fox

**Affiliations:** 1 Department of Cell Biology, Duke University School of Medicine, Durham, NC 27710, USA; delisa.clay@duke.edu; 2 Department of Pharmacology and Cancer Biology, Duke University School of Medicine, Durham, NC 27710, USA

**Keywords:** DNA damage, model organisms, cell cycle, DNA repair

## Abstract

Genome damage is a threat to all organisms. To respond to such damage, DNA damage responses (DDRs) lead to cell cycle arrest, DNA repair, and cell death. Many DDR components are highly conserved, whereas others have adapted to specific organismal needs. Immense progress in this field has been driven by model genetic organism research. This review has two main purposes. First, we provide a survey of model organism-based efforts to study DDRs. Second, we highlight how model organism study has contributed to understanding how specific DDRs are influenced by cell cycle stage. We also look forward, with a discussion of how future study can be expanded beyond typical model genetic organisms to further illuminate how the genome is protected.

## 1. DNA Damage Responses: A Brief Overview 

To protect the genome from damaging events or agents, organisms have evolved DNA Damage Responses (DDRs). DDRs involve several factors that sense DNA damage, signal that damage to the cell, and recruit downstream effector proteins. These effectors then induce cell cycle arrest, repair the DNA damage, or activate processes such as cell death or senescence [1,2,3,4]. 

DDRs can be molecularly distinct, and often depend on the damage source. Inside the cell, DNA damage can arise from changes in metabolism or immune responses, or errors in DNA replication and recombination [1,5]. Outside of the cell, genome damaging agents include ultraviolet (UV) radiation from the sun, ionizing radiation (IR), and chemical drugs often used to treat cancer such as cisplatin or mitomycin C (MMC). These DNA damage sources generate single- or double-strand DNA breaks (SSBs or DSBs) [2]. For simplicity, this review will focus primarily on DSB responses.

DDRs are molecularly complex and intricate, and numerous reviews cited herein cover distinct DDR mechanisms in depth. Here, our goal is to highlight examples of the important role that model organisms have played in understanding DDR regulation during distinct cell cycle events. Within that context, please note that the literature cited is meant to serve as an example rather than a comprehensive overview. We apologize for our omission of important work relevant to this topic that we are unable to cite due to space constraints.

## 2. The Role of Genetic Screens in Model Organisms to Reveal DDR Genes

The foundation of experimental approaches to reveal DDRs arguably derives from early studies in *Drosophila* using genome damaging mutagens such as X-rays [6]. Early pioneering work revealed the existence of DDRs, including damage-induced cell cycle arrest in the ciliate *Paramecium* and the urchin *Arbacia* [7,8] and chromosomal repair events in the plants *Tradescantia* and *Zea Mays* [9,10]. In the 1960s, the first radiation sensitive mutant strains were identified in the budding yeast *Saccharomyces cerevisiae* [11,12]. This was followed by seminal work using radiation sensitive (*rad*) yeast mutants to demonstrate that cell cycle checkpoints are required to survive DNA damage [13].

Model eukaryotic organisms such as *S. cerevisiae*, the fission yeast *Schizosaccharomyces pombe*, the nematode *Caenorhabditis elegans*, the fly *Drosophila melanogaster (*hereafter: *Drosophila)*, and others have contributed greatly to identifying DDR genes. Ample molecular and genetic tools and early sequencing of model organism genomes (near the turn of the 21st century) facilitated genetic screens for DDR genes. The premise of such screens, based on prior successful approaches in *Escherichia coli* (for example see [14]) is that survival after a damaging agent such as a chemical mutagen or irradiation will be compromised in a strain where a DDR gene is malfunctioning. Such screens, using both forward and reverse genetic approaches, revealed numerous DDR genes in yeast, frequently named *rad* (radiation sensitive), *hus* (sensitive to the DNA synthesis inhibitor hydroxyurea), *mec* (mitosis entry checkpoint), or *dun* (DNA damage un-inducible) mutants [15,16,17,18,19]. These screens greatly facilitated study of critical and conserved DDR regulators discussed later in this review, such as *S. cerevisiae* MEC1, ortholog of human ATR kinase (see the section “*Kinase signaling in response to DSBs*”). Beyond genetic screens focused on cell survival, DDR genes can be identified using assays that measure the level of genomic instability, such as altered rates of recombination, loss of a heterozygous marker, genomic rearrangements, or elevated levels of DDR-induced transcription. Use of such assays has continued to fuel DDR genetic screening efforts in yeast [20,21,22,23,24].

Similar screening efforts have been conducted in C. elegans [25,26]. As discussed in the section “*Meiotic DDR regulation*” the germline tissue of this transparent nematode has provided a powerful DDR gene identification system (also see [27,28,29]). An example of an easy DDR-related germline phenotype to screen for in C. elegans is a high incidence of males (him) phenotype, caused by defects in meiosis that generate XO males instead of XX hermaphrodites [30]. Such mutants have been especially valuable in unraveling mechanisms of meiotic DDRs (see the section “*Meiotic DDR regulation*”).

The fly *Drosophila* is another powerful DDR screening model, and animal survival-based screens have identified mutagen sensitive (*mus*) strains, which are mutant for many DDR genes [31,32,33,34]. Such mutants include *mus308*, corresponding to the *polQ* gene, which encodes a major regulator of alternative end joining repair (see the section *DNA repair pathways*”). More sensitive DDR assays have also been employed in *Drosophila*, including DNA repair assays that can be easily linked to eye and bristle phenotypes, as well as a host of cytological assays (reviewed by [35]). In addition to the use of chemical mutagens and irradiation as experimental sources of DNA damage, site-specific DSB systems including CRISPR/Cas9 have been used to great effect to study *Drosophila* DDRs [36,37]. Additionally, our group has exploited the salt excretion function of *Drosophila* rectal papillar cells to screen for DDR genes [38,39,40], which relies on a mechanism to respond to DNA breaks in mitosis (discussed in the section “*The DDR during mitosis*”). By combining site-specific DSBs with feeding animals a high salt (NaCl) diet, mutants that specifically impact mitotic DSBs can be identified in *Drosophila* papillar tissue.

In addition to flies, worms, and yeast, other model genetic systems including the flowering plant *Arabidopsis thaliana* [41,42], and the mouse *Mus musculus* [43] have also contributed to DDR genetic screening efforts. In addition to genetic screens, the egg extract of the clawed frog *Xenopus laevis* has provided a powerful biochemical system to reveal DDR mechanisms [44]. From these efforts and parallel studies in cultured human cells, highly conserved DDRs have been revealed. Such high conservation highlights the clear importance of model organism DDRs to the study of human diseases related to genome integrity. In addition to this high conservation, DDR differences across evolution as well as within specific tissues of the same organism have revealed insight into a critical question- following DNA damage, how does a cell determine which DDR to activate?

## 3. Major Players in DDR Mechanisms: Conservation and Differences across Model Systems

**Figure 1:**
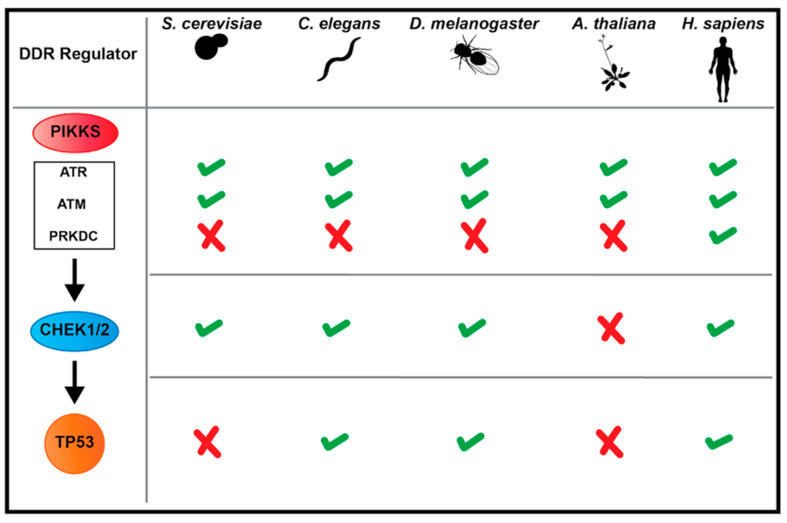
Conservation and distinctions among major DNA Damage Response (DDR) players. Model organisms contain many of the major DDR players found in humans, but also lack clear homologs in specific instances. The human gene names are indicated on the left. Check mark = present in that organism, X = missing in that organism. PIKKS= phosphoinositide 3-kinase (PI3K)-related kinases.

This section discusses some major players that coordinate DDRs, highlighting organism-specific differences where appropriate (Figure 1). As nomenclature between organisms can be confusing, we use the human gene/protein name unless noted otherwise. Due to space limitations, we focus primarily on DSB (not SSB) responses.

### 3.1. Kinase Signaling in Response to DSBs

DSBs are deleterious in nature as they can lead to loss of large amounts of genetic material during cell divisions if not responded to properly [3,45]. DSBs can be caused by sources such as irradiation (X-ray, γ, etc.), or errors in replication in which stalled replication forks collapse into DSBs. DSBs initiate a signal transduction cascade that promotes DNA damage sensing and cellular outputs such as transcription and recruitment of downstream effector proteins for cell cycle arrest, DNA repair, cell death, or senescence. The type of DNA damage can dictate which signaling factors are recruited early on. A great extent of this signaling is driven by 3 phosphoinositide 3-kinase (PI3K)-related kinases (PIKKs). The PIKK ATM- and Rad3-related (ATR), is recruited to aberrant single stranded DNA (ssDNA) that arises during errors in replication. Two other PIKKs, Protein Kinase DNA-activated catalytic subunit (PRKDC) and Ataxia telangiectasia mutated (ATM), are often recruited to sites of DSBs. PRKDC is notably absent in many model organisms (Figure 1) including S. cerevisiae, S. pombe, C. elegans, Drosophila and A. thaliana [46,47,48] (see the sections “Interphase: cNHEJ or HR” and “Emerging model DDR systems”).

ATM and ATR amplify signaling cascades through direct and indirect phosphorylation of downstream substrates. Key players in DDR cell cycle control, the Checkpoint kinases 1 and 2 (CHEK1 and CHEK2), are phosphorylated by ATR and ATM, respectively and are then activated to phosphorylate downstream substrates. ATR-mediated activation of CHEK1 can inhibit Cyclin Dependent Kinases (CDKs) which also leads to cell cycle arrest [46]. All 3 PIKKs can phosphorylate H2Ax (γH2Ax), a histone variant that once activated can recruit downstream factors to repair foci that amplify the DDR signal [2]. ATM and CHEK2 can both regulate the TP53 transcription factor. TP53 is a tumor suppressor with several important DDR functions, but primarily regulates cell cycle arrest, cell death, or senescence [1,2,46]. As discussed in the section “Interphase: cell cycle arrest or apoptosis”, some model organisms lack clear homologs of CHEK kinases or TP53 yet accomplish responses like those carried out by TP53.

### 3.2. DSB Repair Pathways

DSB repair is typically coordinated through a signal transduction cascade that mediates cell cycle arrest to allow for DNA repair before continuing the cell cycle. Failure to properly coordinate cell cycle checkpoints with DNA repair can result in persistent DNA damage and may eventually lead to loss of genetic information during cell division, or to mitotic catastrophe, a form of cell death that arises from mitosis [49]. To prevent such problems, two major repair pathways respond to DSBs: Homologous Recombination (HR) and Classical Nonhomologous End-Joining (cNHEJ). Other DSBs repair pathways that are gaining increasing attention are Alternative End-Joining (alt-EJ, also known as Theta-Mediated End-Joining, TMEJ or Microhomology Mediated End-Joining MMEJ), and Single-Strand Annealing (SSA) [2,50,51].

HR is an error-free mode of repair which involves the processing of DSB ends through short and long-range resection with various endo- and exonucleases. RAD51 is recruited to resected ssDNA and drives the search for a homologous template to allow for accurate repair. cNHEJ is an error-prone form of DSB repair that involves the Ku family proteins (XRCC5 and XRCC6) binding to either side of the DSB. Ku proteins then recruit a host of factors such as PRKDC and enzymes that process DNA ends, and the processed ends are ligated together. Alt-EJ and SSA have similar modes of repair with distinct machinery. During alt-EJ, which primarily depends on DNA polymerase theta (POLQ) in *C. elegans*, D. melanogaster, A. thaliana, and vertebrates, microhomologies around the site of a break are recognized and annealed together [37,52,53,54,55,56,57,58,59,60]. In yeast, similar microhomology-based repair (MMEJ) requires the polymerases Pol4, Polζ, and Polη [61,62]. For SSA to occur, longer regions of homology are required for annealing. The single strand annealing protein RAD52 is thought to be required for this process in humans as well as in *S. cerevisiae*, and *S. pombe*. RAD52 is missing in Drosophila, though other factors have been hypothesized to be involved such as RecQ5 and Smarcal1 in Drosophila as well [2,35,50,63,64]. Having introduced some of the major DDR players, we will next discuss how cell cycle state can determine which DDR occurs.

## 4. DDR Responses During the Mitotic Cell Cycle Across Model Systems

DNA damage necessitates a series of decisions. Arrest the cell cycle or initiate cell death? Repair by HR, cNHEJ, or another pathway? Specific DDRs underlie each decision and are impacted by the molecules available and the specific state of the damaged cell. Insights from model organisms have played essential roles in revealing how conserved DDR players function and how DDR decisions are shaped by evolution.

### 4.1. Interphase: Cell Cycle Arrest or Apoptosis

**Figure 2:**
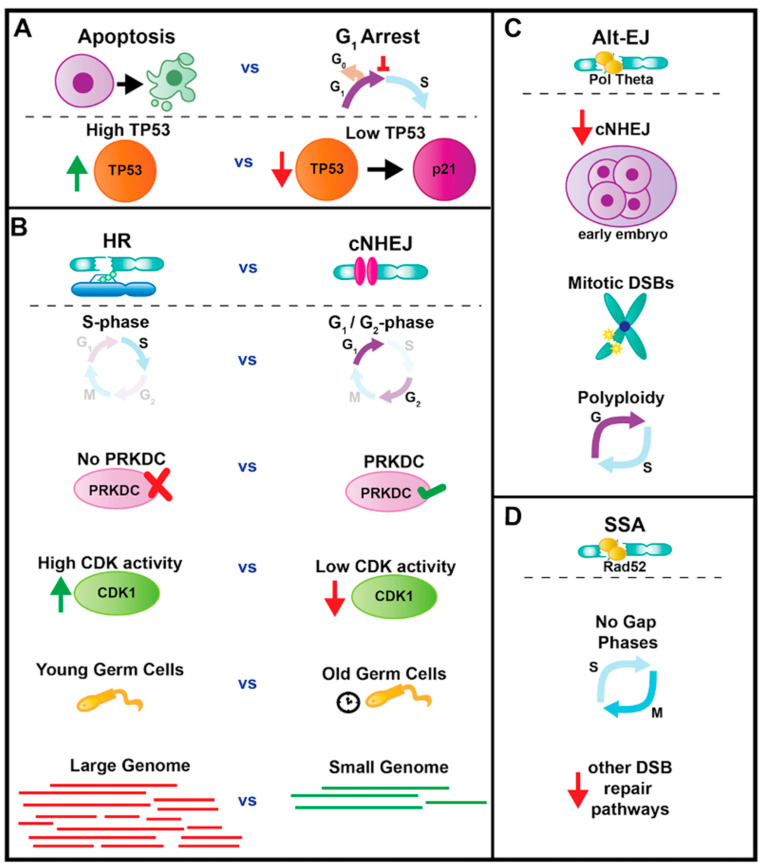
Factors impacting key DDR decisions. (**A**) The level of p53 protein plays an important factor in the cell cycle arrest vs. apoptosis decision. (**B**) Phase of the cell cycle, presence of PRKDC, levels of CDK1, age of germ cells, and genome size all play a role in the HR vs. cNHEJ decision for DSB repair. (**C**) Examples of high alt-EJ activity are in the early embryo of some organisms, as well as during mitosis and in polyploid cells. (**D**) Examples of high SSA activity are in cells cycling without gap phases and when factors for other DSB repair pathways are decreased.

TP53 orthologs (hereafter p53) play recurring roles across evolution in activating DNA damage-induced apoptosis. In vertebrates, p53 plays an additional role in activating cell cycle arrest in response to DNA damage. p53-mediated arrest occurs through inhibition of the G1/S cyclin kinase inhibitor P21/CDKN1A (Figure 2A) [65]. Divergence among p21 family members may explain why p53 does not induce a robust cell cycle arrest in Drosophila and *C. elegans* [66,67,68]. In vertebrates, differing levels of p53 appear to contribute to the apoptosis or cell cycle arrest decision (Figure 2A), which may reflect the difference between transient and sustained DNA damage [69,70,71]. The absence of a p53 ortholog in yeast and the lack of a p53-mediated cell cycle arrest in Drosophila and *C. elegans* may explain why DNA damage-induced G_1_ phase arrest is not as prolonged when compared to mammalian cells [72,73,74]. It is interesting that p53 function appears to increase across evolution as organismal cell number increases. Recently, elephants, which are highly cancer resistant, were found to have an astounding 20 copies of TP53 [75,76].

Compared to p53, CHEK1 and 2 kinase orthologs play more widely conserved roles in mediating cell cycle arrest. These kinases, which are activated by PIKKs, are especially important in arresting the mitotic cell cycle at the G_2_/M transition in response to DNA damage. This arrest occurs through regulation of CDC25 phosphatases, which in turn control activity of the mitotic CDK [77,78]. A. thaliana contains ATM and ATR homologs but lacks both CHEK kinases and p53. Instead, the SOG1 transcription factor responds to ATM and ATR activity to direct cell cycle arrest and other DDRs [42,79,80,81]. A recent study [81] found that TP53 and SOG1 have many overlapping classes of gene targets, yet SOG1 also plays an important role in responding to fungal pathogens, which could provide a clue for the distinct DDR wiring between plants and animals.

### 4.2. Interphase Repair: cNHEJ or HR

Cell cycle stage can dictate repair pathway choice, as a particular cell cycle stage has distinctions in availability of a homologous template, chromatin organization, and protein abundance [82,83]. It had previously been hypothesized that during G_1_-phase of the mitotic cell cycle, cNHEJ predominates as the preferred DSB repair pathway. Then, when a sister chromatid is generated during S-phase, it was speculated that HR becomes the pathway of choice from S-phase to G_2_ [84,85]. In contrast to this view, in 2005, Mao et al. [85] used the site-specific endonuclease I-SceI to induce DSBs and fluorescent reporters of HR and cNHEJ repair in non-cancerous human cells. They reported that cNHEJ is active throughout the cell cycle. HR predominates during S-phase and declines during G_2_, in favor of cNHEJ (Figure 2B). This updated view of repair pathway choice plays important roles in current efforts to employ CRISPR/Cas9 for genome editing [86,87].

The preference of cNHEJ over HR appears to be shaped by the organism. In organisms with highly repetitive genomes like humans, erroneous yet rapid cNHEJ may be preferred over the slower HR process [85]. Indeed, mammalian cells are much less sensitive to loss of HR machinery after DSBs compared to E.coli [88,89]. In other organisms, some aspects of cNHEJ machinery appear to be missing. This appears to be reflected in a decrease in cNHEJ efficiency in S. cerevisiae and S. pombe, which lack PRKDC but have Ku proteins (Figure 2B). However, Drosophila and other insects also appear to lack PRKDC, but carry out efficient cNHEJ, suggesting evolutionarily divergent mechanisms may drive cNHEJ [35,90]. Yeast also lack poly (ADP-ribose) polymerase, or PARP, a major regulator of multiple DNA repair processes in eukaryotes [2].

HR does play an important role in DSB repair during DNA replication. During mammalian cell S-phase, both damage-inducing agents such as UV radiation and replication fork stalling and collapse cause single-ended DSBs. As repair of single-ended DSBs via cNHEJ could generate deleterious chromosomal rearrangements, HR is the pathway of choice [89,91]. When HR does occur, intra-chromosomal recombination is preferred to inter-chromosomal recombination, as shown using Drosophila I-SceI-Induced DSBs in genetic backgrounds that differentiate each HR repair outcome [92]. Aside from single-ended DSBs, HR and cNHEJ can compete for DSB repair in S-phase [93]. HR efficiency during cell cycle stages is also regulated by CDK activity. As CDKs are required for the phosphorylation of key DSB end resection factors, HR is active when CDK activity is higher during S- and G_2_-phases (Figure 2B) [91,94]. HR appears to decline with age in premeiotic germ cells in Drosophila, and this decline is linked to genomic instability and is a likely cause of age-induced infertility (Figure 2B) [95]. There is also evidence that organisms with larger genomes (and thus more genome to replicate per cell), such as mammalian cells, depend more on HR machinery during S-phase than in S. cerevisiae or S. pombe (Figure 2B) [90]. In yeast and Drosophila, homology-directed repair also plays a role following breakage of an aberrant dicentric chromosome. Such breakage is repaired by break-induced recombination, a process involving either homologous recombination or chromosome healing by addition of a new telomere to the broken end [96,97].

### 4.3. Roles for Alt-EJ and SSA Repair

Alt-EJ repair is often described as a “back-up” repair pathway. This is because inactivation of the major alt-EJ regulator POLQ in C. elegans, Drosophila, and mice has minimal impact on survival to several DNA damaging agents, whereas POLQ plays an important role for surviving DNA damage in the absence of cNHEJ or HR factors [37,52,53,54,55,56,57]. However, emerging evidence is revealing important roles for this pathway in numerous contexts. The DNA end resection that occurs during alt-EJ can also serve as a template for cNHEJ, and so it is plausible that alt-EJ should be active throughout the cell cycle (Figure 1A) [93]. In early zebrafish embryo development, the conserved alt-EJ regulator POLQ is required for animal survival following UV irradiation [98]. This reliance on a “backup” repair pathway may be due to decreased availability of key cNHEJ components such as PRKDC, which is expressed at low levels in early embryos of both the zebrafish and mouse (Figure 2C) [98]. In the section “The DDR during mitosis”, we further discuss how alt-EJ can play a prominent role in surviving DSBs.

As SSA requires extensive long-range resection similar to that seen during HR [99], this repair pathway potentially occurs as a back-up to HR during S/G_2_. From studies in yeast (S. cerevisiae and S. pombe), Arabidopsis, chicken, and mammalian cells, SSA appears to be prevalent whenever SSA proteins are available and other DSB pathways are not (Figure 2D). But SSA also can function as a primary repair pathway in some instances. Similar to the dependence on alt-EJ during early development in zebrafish, Drosophila blastoderm cells of the embryo, which are finishing rapid cell cycles that lack gap phases, appear to depend on SSA (Figure 2D). This reliance eventually switches to cNHEJ repair as embryogenesis proceeds (and gap phases are introduced) [100]. Going forward, further study of rapid cell cycles of cells from early embryos is likely to reveal unique DDRs. For example, work on cultured mouse embryonic stem cells, which have very short G1 phases, found evidence of continued cell cycling in the presence of massive ssDNA gaps. Succesful cell cycles in this context requires ssDNA-binding DDR regulators such as ATR and RAD51 [101].

### 4.4. The DDR During Mitosis

Much of what is known about the DDR, including DNA repair, is in the context of interphase cells. But DNA damage present during mitosis also poses a threat to genome integrity. Mitotic DNA damage can lead to mis-segregation of whole chromosomes or chromosome fragments [102]. This can lead to aneuploidy or micronuclei, a nuclear atypia implicated in cancer initiation and progression. Increasing evidence has shed light on how cells tolerate DNA damage present during mitosis. It is now understood that mitotic cells can activate aspects of interphase DDRs. In particular, both repair protein recruitment and DNA repair synthesis have been observed to occur during mitosis [103,104,105,106].

In human cells, several factors involved in mediating interphase DDRs are also important for mitotic progression. While many early aspects of DNA repair appear active during mitosis of human cells, the mitotic kinases CDK1 and Polo Like Kinase 1 (PLK1) work during mitosis to block recruitment of downstream repair factors known to participate in HR and cNHEJ [105,107]. For the most part, upstream DDR signaling during mitosis in human cells is similar to what is observed during the G_2_/M checkpoint. PIKKs are active and recruit early-acting repair factors. This includes the MRN complex, which functions as a DNA damage sensor and endonuclease [108,109]. However, beyond these upstream events, many further downstream DDR signals are not known to be active in mitosis, perhaps because the presence of specific repair intermediates may interfere with chromosome segregation. However, repair regulation still plays important roles during mitosis. For example, broken human chromosome fragments can be tethered together through filaments involving the DNA repair scaffold MDC1 and the repair mediator TOPBP1 [110].

Model organism studies have also revealed important regulation of DSBs in mitosis. S. pombe cells can generate neocentromeres on broken DNA to promote mitotic segregation [111,112]. In Drosophila, genome instability is also prevented when DNA damage is present during mitosis. In Drosophila neuroblasts, Polo kinase and the SAC proteins BubR1 and Bub3 form tether structures that enable broken chromosome fragments during mitosis to segregate into daughter nuclei [113,114,115,116,117]. Our group has identified a mechanism used by Drosophila papillar cells to similarly transport broken chromosome fragments into daughter cells during mitosis. Papillar cells are polyploid (see “DDRs during and after endoreplication cycles”) and lack a requirement for p53 or CHEK family kinase signaling in response to DSBs. Instead, chromosome fragment segregation in this cell type relies on the alt-EJ regulator PolQ and members of the conserved Fanconi Anemia DNA repair family (Figure 2C) [39,40]. Our findings may mirror those of mammalian cells, where Fanconi Anemia proteins localize to regions of the genome that are sites of mitotic DNA synthesis (MIDAS) [118]. Such regions contain ultra-fine DNA bridges [119], which may link chromosome fragments created by DSBs. Further, our identified role for PolQ in mitosis is relevant to human cancer, as cancer cells lacking the BRCA2 or RAD52 repair proteins rely heavily on PolQ-mediated alt-EJ repair in mitosis (Figure 2C) [120]. Taken together, it is clear that mitotic cell cycle stage, developmental stage, cell type, and organism type all influence DDR responses.

## 5. DDR Responses during Other Cell Cycles in Model Systems

### 5.1. Meiotic DDR Regulation

In addition to the mitotic cell cycle, variant cell cycles are conserved across evolution. Meiosis is distinctive in that DSBs are part of a programmed process, the goal of which is to generate genetic diversity. HR is the preferred pathway of choice in meiosis, as it generates cross-over exchanges between homologs. Therefore, HR dominates in prophase I of meiosis. The HR regulator Rad51 and other Rad51-related proteins such as DMC 1 (Humans, S. cerevisiae, A. thaliana) or Spindle-d (Drosophila) drive meiotic DSB repair between homologous chromosomes in diverse systems [121,122,123]. In the C. elegans germline, the ATM homolog ATM-1 also plays an important role in ensuring that HR occurs between the homologous chromosome rather than the sister chromatid, which promotes genetically diverse gametes [124]. Recently, Macaisne et al. [125] showed that when HR is compromised in the him-5 mutant C. elegans germline, then alt-EJ and SSA function redundantly to repair DSBs. At the same time, the C. elegans TP53 homolog CEP-1 suppresses Ku family cNHEJ proteins [126]. Later in meiosis, and similar to the above discussion of broken chromosome regulation in mitosis, broken meiotic chromosome fragments can segregate by poleward microtubule-generated forces in C. elegans oocytes [127]. These studies highlight how DDRs can be adapted during meiosis to ensure successful gamete formation. For more on the extensive topic of meiotic DSB repair and the role of model organisms, we refer readers to recent reviews [128,129,130].

### 5.2. DDRs during and after Endoreplication Cycles

In addition to the meiotic cycle in germ cells, somatic cells also possess a common cell cycle variation known as the endoreplication cycle. Endoreplication involves copying genomic content more than once without an intervening cell division. This generates a whole genome duplication, or polyploidy. Once considered a process restricted mainly to specific plant and insect tissues, endoreplicated polyploid cells have now been identified in nine of eleven human organ systems, most notably in the mammalian placenta, liver, and heart, and skeletal muscle [131,132,133]. Polyploidy can be developmentally programmed or arise from external environmental stresses such as tissue injury or DNA damage. Indeed, several forms of DNA damage have been observed to result in polyploidization [134,135,136,137]. Aberrant polyploidy underlies disease, including in an estimated 37% of all human cancers [138], and polyploidy can arise from extended exposure to the chemo-sensitizing agents [136].

During endoreplication, cells either undergo cycles of G and S phases (known as endocycles) or enter into mitosis without completing cell division (endomitosis). Endoreplication can result in mono- or multi-nucleate polyploid cells depending on whether nuclear division has occurred. Cells can also become polyploid through cell-cell fusion, which can give rise to multi-nucleate cells [131]. While we have previously discussed connections between DNA damage and polyploidy [139,140], here we provide an update on this important topic.

Following DSBs in A. thaliana, the TP53 analog SOG1 promotes endocycles in dividing meristematic cells, whereas SOG1 instead promotes apoptosis in other cell types [80,141]. The mechanisms underlying this cell-type specific, DDR-induced polyploidy remain unclear, though work from tissue injury studies in Drosophila may offer a clue. Baseline expression of the Drosophila fizzy-related gene can predispose specific tissues to endocycle upon injury stress [142,143,144]. The A. thaliana fizzy-related homolog, CCS52A1, is a SOG1 target, and it will be interesting to investigate if tissue-specific differences in expression of this gene underlies DDR-induced polyploidy in plants.

As we have reviewed previously [140,145], polyploidy that results from endocycles often leads to local fine tuning of genome copy number, which can enable new gene expression states. For example, rather than even duplications of all genome segments, specific regions can be under-replicated or locally amplified in the polyploid genome. Such under-replication and gene amplification during endocycles can generate DSBs [146]. Recent work has explored DDR regulation following endocycles further. In Drosophila polyploid follicle cells, Alexander et al. investigated a role for the alt-EJ gene polQ, in DSB repair at chorion genes, which are a discrete amplified region of the follicle cell genome. In response to DSBs that arise from chorion gene amplification, alt-EJ, cNHEJ, and HR all compete for repair. The DSB pathway of choice appears to be dictated by genomic locus, underscoring the importance of cellular context in DSB repair pathway choice [147].

To enable tolerated DSB events, components of DDRs can be suppressed in polyploid cells. In Drosophila salivary glands, endocycling cells suppress apoptosis by epigenetic silencing of pro-apoptotic genes at the H99 locus and downregulation of p53 isoforms [148,149]. In the Drosophila fat body, an adipose tissue, ATM is downregulated to prevent sensing of DSBs generated by under-replication, and this mechanism is tied to adipose tissue growth [150]. While the presence of DSBs explains why endocycling cells downregulate DDR components, DDR gene inactivity also occurs in polyploid cells formed by other mechanisms. For example, mammalian cardiomyocytes and hepatocytes downregulate apoptotic genes after becoming polyploid [151]. Overall, recent progress suggests that endoreplication may render the resulting polyploid cells less sensitive to genome damage. Such altered DDR states of polyploid cells is likely important to diseased states, where polyploidy accumulates aberrantly. For example, aberrant polyploidy in Drosophila neural stem cells generates DNA damage [152]. In apoptosis-resistant transformed human cells, Chitikova and colleagues [153] demonstrated that X-Ray IR-induced polyploidy is accompanied by delayed recruitment of the cNHEJ protein TP53BP1, which was sustained for several days following IR. Such findings may explain radiation resistance in cancers with polyploidy. The increasing appreciation of both natural polyploidy in diverse organisms and the role of polyploidy in disease will likely reveal future connections between polyploidy and DDRs.

## 6. Emerging Model DDR Systems

The above sections highlight the immense contribution of model systems to our current understanding of DDRs. However, in this era of whole genome sequencing and CRISPR/Cas9 genome editing, the barriers to studying non-traditional models have been substantially lowered. As a result, we can now incorporate knowledge on DDRs from countless non-traditional model organisms.

Animals of the Tardigrada phylum (tardigrades), for example, exhibit extreme tolerance to various forms of radiation [154,155,156,157,158,159]. This may be due, in part, to tardigrade-unique proteins [160,161], and/or to an expansion of conserved DDR genes. For example, whereas flies and humans contain a single copy of the MRN complex protein MRE11, the tardigrade R. variernatus contains fifteen versions of this protein [160]. The archaeon, Haloferax volcanii, which is a naturally polyploid organism, accumulates DSBs and depends on MRE11 to delay potentially deleterious HR in favor of alt-EJ following DNA damage [162]. Similarly, D. radiodurans, a highly radiation resistant, polyploid bacterium, depends on MRE11 to repair excessive γ-radiation induced DSBs. Interestingly, a DNA polymerase, polymerase X (PolX) was also shown to be important for DSB repair in this system. The effects of MRE11 and PolX deletion are additive, suggesting that back up pathways exist in D. radiodurans to repair excessive DSBs [163].

The reliance on a handful of model organisms can also lead to gaps in our understanding of protein evolution. Our above discussion of the PIKK PRKDC could lead one to the erroneous conclusion that this protein is a vertebrate-specific innovation. However, while Drosophila lacks a clear PRKDC, both mosquito and honeybee have apparent PRKDC orthologs [164], and the slime mold Dictyostelium discoideum requires its PRKDC ortholog for cNHEJ [165,166].

Comparing commonly used model organisms to related, yet less commonly studied models will likely also prove fruitful. For example, since the 1930s it was noticed that it was difficult to recover X-radiation-induced mutants in the fungus gnat Sciara coprophila, due to an increased radiation tolerance compared to Drosophila [167,168,169,170]. The mechanism behind such radiation resistance remains unknown, yet the Sciara genome was recently fully sequenced [171]. Future efforts should endeavor to incorporate a more diverse array of model systems to fill in our knowledge of DDR mechanisms.

## 7. Conclusions

In summary, organisms have evolved conserved DDRs to preserve the integrity of genetic material. Model genetic organisms have played a major part in study of DDRs to date, due in part to the power of genetic screens. The specific DDRs that are activated in a given biological context impact decisions such as repair pathway choice and cell cycle arrest versus apoptosis. Biological context in turn impacts the specific DDR, and here we have specifically highlighted how cell cycle state is critical to selecting which DDR is activated. As work in model genetic organisms has emphasized, such cell cycle states go beyond the mitotic cell cycle, impacting germ cell meiotic cycles and somatic endoreplication cycles as well. In addition to our discussion here, we refer the readers to other excellent reviews on this topic [47,74,172]. Continued study across the evolutionary spectrum, in model organisms and beyond, can continue to answer the fundamental question of how a specific DDR is active in response to genome damage.
